# In this current wildfire crisis, acknowledge widespread suffering

**DOI:** 10.1007/s13280-024-02105-5

**Published:** 2025-01-28

**Authors:** Fiona E. Newman Thacker, Kathleen Uyttewaal, Tomás Quiñones, Rik Leemans, Bethany Hannah, Cathelijne R. Stoof

**Affiliations:** 1 Soil Physics and Land Management Group, Wageningen University and Research, PO Box 47, 6700 AA Wageningen, The Netherlands; 2 Earth Systems and Global Change Group, Wageningen University and Research, PO Box 47, 6700 AA Wageningen, The Netherlands; 3 Research and Development Department, Technosylva, Parque Tecnológico de León, C/ Nicostrato Vela, Edificio Technosylva, 24009 León, Spain; 4 American Wildfire Experience, PO Box 24, Kyburz, CA 95720 USA

**Keywords:** Adaptation, Climate change, Disaster management, Suffering, Wildfire

## Abstract

With climate change causing more extreme weather events globally, climate scientists have argued that societies have three options: mitigation, adaptation or suffering. In recent years, devastating wildfires have caused significant suffering, yet the extent of this suffering has not been defined. To encapsulate this suffering, we determined impacts and effects of extreme wildfires through two systematic literature reviews. Six common themes of wildfire suffering emerged: environmental, social, physical, mental, cultural and resource suffering. These themes varied in scale: from local to regional; from individuals to communities; and from ecosystems to landscapes. We then applied these themes in the Las Maquinas (Chile) and Fort McMurray (Canada) wildfires. This highlighted several adaptation strategies that can reduce suffering, however our exploration indicates these strategies must address social and ecological factors. This analysis concludes that suffering from wildfires is diverse and widespread, and that significant engagement with adaptation strategies is needed if this is going to decrease.

**Supplementary Information:**

The online version contains supplementary material available at 10.1007/s13280-024-02105-5.

## Introduction

In an age of global environmental change, wildfires are growing in their devastation around the world. Despite research indicating that the frequency and burned area of wildfire events are globally decreasing, recent years have seen increasingly common wildfire disasters across almost all regions of the world (Krawchuk et al. 2009; Duane et al. 2021). Fire is often an essential ecosystem process, and the use of traditional, rural and Indigenous fire has a very long history (Christianson et al. 2022; de Oliveira et al. 2023). However, in recent decades, catastrophic fires are now reaching levels in which even people who are prepared to try and defend their homes, are forced to flee last minute only to perish in their escape (Ribeiro et al. 2020). Age-old cultural sites and fire refugia, unburned patches within a wildfire area, have little defense against this new era of wildfire. Beyond the flames, smoke spreads regionally and settles in far-away cities. The associated struggles with air quality emphasises that wildfires do not have to be proximate to cause suffering (Xie et al. 2020; Milman 2023). Suffering from wildfires can become so omnipresent that seasoned firefighters struggle to come to terms with the fires’ impacts, and personally grapple with an endemic of mental health illnesses and substance abuse (Singer 2021). Wildfire disasters are taking an increasing toll on communities alongside the natural environment; and further suffering can be expected in an uncertain climatic future (Turco et al. 2019; Costa et al. 2020; Dupuy et al. 2020; Duane et al. 2021). Understanding that not all fires are ‘bad fires’, there are continued calls to accept and coexist with fire (Moritz et al. 2014; Stoof and Kettridge 2022). Acknowledging that suffering is associated with the impacts of a small but increasing number of damaging events, is crucial to facilitate measures that likely help to mitigate such suffering in the future.

The concept of suffering was originally coined by John Holdren (2008) in his 2007- presidential address of the AAAS (Holdren 2008):

“*Facing the menace of growing, human-caused disruption of global climate, civilization has only three options: mitigation (taking steps to reduce the pace and the magnitude of the climatic changes we are causing); adaptation (taking steps to reduce the adverse impacts of the changes that occur); and suffering from impacts not averted by either mitigation or adaptation. We are already doing some of each and will do more of all, but what the mix will be depends on choices that society will make going forward.*”

Despite development of significant climate mitigation and adaptation actions, Holdren’s words remain pressingly true. Popular media often uses images of wildfires to illustrate the impacts of climate change on natural hazards. However, applying the concept of suffering to wildfire events is vastly unexplored, even as each year changing and novel fire regimes challenge regions’ ability to cope with such events. Most studies instead focus on assessing vulnerabilities, risks and impacts. Although significant amounts of research have uncovered the danger in only investing in reactive measures to wildfires (Kauffman 2004; Otero and Nielsen 2017; Castellnou et al. 2019), more proactive strategies to prevent wildfire disasters remain challenging to initiate and sustain (Miller et al. 2020; Bacciu et al. 2022; Copes-Gerbitz et al. 2022). Often significant action is only triggered by a large disaster, such as the 2017 Pedrógão Grande fires (Portugal) which killed over 60 people, and resulted in the founding of the Agency for the Integrated Management of Rural Fires (AGIF) (Alcasena et al. 2021). The lack of a comprehensive evaluation of the full range of impacts from wildfire disasters, alongside continued focus on fire suppression and the direct aftermath of wildfires can contribute to the challenge of creating effective and lasting wildfire governance.

Therefore, acknowledging the full scope of suffering from wildfire disasters helps to clarify the crucial need for wildfire adaptation and directly facilitates such adaptation. The use of the term ‘suffering’, defined by *The Oxford English Dictionary* as “the state of undergoing pain, distress or hardship”, can be used to explore the impacts from fires, beyond the statistics and categorizations that are often used to characterise vulnerabilities. Research has shown that various predefined vulnerabilities, including gender, race and economic status, are pre-conditions that amplify the likelihood of suffering in wildfire disasters (Palaiologou et al. 2019; Méndez et al. 2020; Lambrou et al. 2023), whereas vulnerability is projected, what *might* happen in a particular context, suffering is experienced, what *did* happen. These are rarely the same thing—projections of impacts are not always cohesive; some are complex to measure; and the art of precisely predicting wildfires is notoriously difficult (Fairbrother and Turnley 2005; Ager et al. 2021).

Focusing on suffering rather than vulnerability or adaptability likely humanises the impacts of wildfires, encapsulating the impacts on real people, animals (fauna), vegetation (flora) and infrastructures, rather than reconstructing them as numbers and statistics. Moreover, the concept of suffering transcends a western scientific focus on “impacts” as it has spiritual, emotional and psychological implications. Addressing suffering forms the bedrock of major world religions like Buddhism, Christianity, Hinduism, Judaism, Hinduism and Judaism (Lewis Hall and Hill 2019). Understanding suffering is moreover a pillar of psychology and philosophy, where scholars like Kierkegaard argue that suffering is a key part of forming human identity (Cuff Snow 2016). In this analysis, we propose six distinct ‘themes of suffering’ from wildfire disasters. We predominantly focus on extreme wildfires where the suffering is widespread, but we also acknowledge that suffering occurs even when wildfires are not labelled a ‘disaster’. We apply this framework of suffering to two wildfire disasters in Canada and Chile, and interweave current knowledge on wildfire adaptation strategies to identify actions which may mitigate some of the associated suffering.

## Methodology

To explore the themes of suffering associated with wildfire events, we conducted two systematic literature reviews. We first produced an overall analysis of the current status of wildfire research through the concept of suffering (Review 1), and then reviewed how impacts and effects from extreme wildfires could be themed using the concept of suffering (Review 2). These systematic reviews were conducted following RepOrting standards for Systematic Evidence Synthesis (ROSES) criteria, a reporting standard developed for environmental management and conservation (Haddaway et al. 2017). Below, we summarize the protocol, in depth details are provided in the Supplementary information 1.

### Search strategy and outputs

The search strategy differed between Review 1 and Review 2 and both searches were conducted solely in English. For Review 1, the search string “wildfire, suffering” was used in Web of Science and SCOPUS between the dates 1990–2022. In Review 2, the keywords “extreme wildfire impacts” and “extreme wildfire effects” were used in Web of Science between the dates 2013–2023. The word ‘extreme’ was used to narrow the outputs of Review 2 to where the major impacts are likely to be indicated. The comprehensiveness of the search was estimated with 13 articles, of which 8 were included within the output of these two reviews (Table S1).

The search outputs for Review 1 resulted in 270 articles across both databases (*n* = 245 after duplicates). In Review 2, 1198 articles were extracted. After removing duplicates when compared with Review 1 and across the two search terms (impacts, effects), 899 articles remained.

The title and abstract of each paper were then screened using nine exclusion criteria, ensuring focus on post-fire impacts on environmental and societal processes. Therefore, papers were excluded if they focused on:

- Prediction of wildfires (risk assessment, ignition probability, wildfire spread modelling)
- Drivers of wildfires with no study of impact
- Evaluation of management techniques on wildfire outcomes
- Irrelevant temporal or spatial scale (paleofires, atomic behaviour, laboratory experiments)
- Evaluation of methodologies/technologies with no link to wildfire impacts
- Policy evaluation
- Impacts of other natural hazards/environmental risks (mention of wildfire but no direct study of wildfire impacts)
- Retraction or Comment on previous paper
- No discernible link to wildfires

The breakdown of the number of papers excluded per criterion is shown in Table S2.

Altogether, the database search, title and abstract screening resulted in 108 papers for Review 1, and 425 papers for Review 2. For transparency, results from articles that concentrated on extreme weather events while explicitly mentioning wildfires (*n* = 46) are shown separately from the main outputs that particularly concentrate solely on wildfires. The included articles and associated data can be viewed in the Supplementary information.

### Narrative synthesis

Narrative synthesis, as described by Popay et al. (2006, p.5) as “an approach to the systematic review and synthesis of findings from multiple studies that relies primarily on the use of words and text to summarize and explain the findings”, was used to analyse the results from Review 1 and 2. Firstly, we aimed to narratively synthesise the outcomes from Review 1 and comprehensively theme post-wildfire suffering. These themes were not based on the empirical findings of the individual papers reviewed (quantitative or qualitative) but rather on the overall research topic and the aims of the article. From the synthesis of the outcomes from Review 1, five themes of suffering were identified: environmental, physical, mental, social and resource suffering. Further to these core themes of suffering, we additionally included articles that addressed positive impacts of wildfires and placed these in a separate category.

Using these five themes from the narrative synthesis of Review 1, the outcomes from Review 2 were analysed and categorised. Whilst the majority of research articles included in Review 2 could be categorised using the five themes already produced from Review 1, several articles did not fit these predetermined themes. Therefore, an additional category of cultural suffering was created to classify these outputs.

## Categorising suffering from wildfire events

**Fig. 1:**
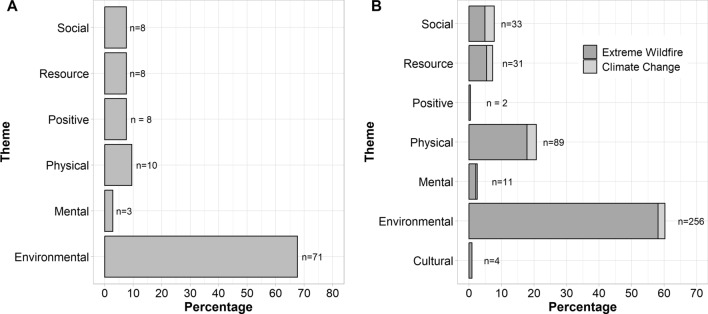
**a** breakdown per theme of suffering from a literature review encompassing research from 1990 to 2023 specifically regarding suffering and wildfire, indicating the percentage of each theme and the number of articles; **b** summary of a literature review exploring recent (2013–2023) extreme wildfire impacts and effects, split by whether the article pertained to wildfires explicitly or climate change induced extreme weather events, and displayed in percentage along with number of articles. An additional theme was added from this second literature review to account for cultural suffering

From the results of the literature reviews, and the resulting six themes of suffering (environmental, physical, mental, social, resource and cultural), it is clear suffering from wildfires encompasses both environmental and anthropogenic consequences from wildfires. The status of current research surrounding suffering from wildfires, alongside characterising their impacts and effects within a framework of suffering, is shown in Fig. 1. The derived themes are not hierarchised; one type of suffering is not inherently more important or more impactful than another kind. Using these six themes of suffering as a framework, we then explore each in greater depth, alongside discussing how wildfires may also create positive impacts.

### Environmental suffering

By a large extent, the most concentrated focus in the studied literature was on environmental suffering (61%). Environmental suffering from wildfires is widespread, including but not limited to, negative impacts on soil (Depountis et al. 2020; He et al. 2021), microbes and fungi (Buscardo et al. 2015; Whitman et al. 2019; Moura et al. 2022), permafrost (Munkhjargal et al. 2020) and atmospheric pollution (Alifa et al. 2020). Hydrological processes are also often adversely affected by wildfires. For example, chemical compositions within watercourses can be altered (Johnston and Maher 2022), alongside negative effects on some water-based biota (Cooper et al. 2021; Kremer and Caldwell 2022; Lawrence et al. 2022). Particle composition and consequent biogeochemical processes within oceans have also been shown to be temporarily altered by large wildfires with ecological consequences (Li et al. 2021; Ardyna et al. 2022). Wildfires have been documented to cause suffering to both wildlife and livestock (Ancillotto et al. 2021; Cowled et al. 2022; Gomez Isaza et al. 2022), and can cause localised species extinctions (Potvin et al. 2017). Even populations of species that are described as ‘fire resilient’, such as the Mediterranean species *Pinus pinaster* and *Pinus halepensis* (Vilà-Cabrera et al. 2012), can still suffer extensively with altered fire regimes that are characteristic of climatic change. This is particularly prevalent when wildfires happen at frequencies such that the initial regrowth does not have time to mature, and so causes change in species composition and structural simplification in the area (Eugenio and Lloret 2004; Eugenio et al. 2006). These examples indicate how even systems that need fire to be ecologically successful, are being overwhelmed and pushed out of balance by a new era of wildfire (Sánchez et al. 2021; Silvério et al. 2022).

### Physical suffering

Wildfires are also capable of producing physical suffering (e.g. health impacts), which is acknowledged within the literature as the second highest topic in both reviews (18%). Both civilians and first responders can experience physical suffering from wildfire events. Direct contact with the flames can result in burns, from minor injuries to severe life changing burns, and death (Bowman et al. 2019). Furthermore, people can perish from fires due to vehicle accidents (Blanchi et al. 2014; Haynes et al. 2020), heat stress and suffocation (Molina-Terrén et al. 2019). First responders can die flying aerial attack planes (Butler et al. 2015; Molina-Terrén et al. 2019) as well as working on the ground (Haynes et al. 2020). In recent decades, high death tolls have been suffered in many countries, including, but not limited to, Australia (2009, 173 fatalities (Hansen 2018)), the U.S.A (2023, 97 fatalities (Hassan 2023)), Greece (2018, 103 fatalities (Vallianou et al. 2020)) and Algeria (2021, 69 fatalities (Ouzou 2021)).

Physical suffering also extends beyond the direct fire line, carried by the smoke and emissions associated with these events. In the short term, exposure can exacerbate respiratory conditions such as asthma and COPD (Bowman et al. 2019; Aguilera et al. 2020), and can particularly impact vulnerable populations such as children (Holm et al. 2021) and the elderly (Youssouf et al. 2014). Inhalation of particulates can also cause increased mortality within firefighters and civilians (Tarín-Carrasco et al. 2021), but often the full effects are not immediately apparent, for example, cancers in firefighters can take years to develop (Stec et al. 2018; Wolffe et al. 2022). The initial impacts of smoke inhalation, such as decreased lung function, do not always dissipate with the smoke, and can go on to have consequences for years after (Orr et al. 2020). Likewise, the great spatial reach of wildfire smoke can have health implications for populations outside the proximate area affected by the flames, indicating the complexity in fully understanding the true impacts of wildfire emissions in terms of physical suffering (Xie et al. 2020).

### Social suffering

Large wildfires, and particularly wildfire disasters, can cause the breakdown of social structures such as community groups, destroy social infrastructure such as homes (Syphard et al. 2012; McKinnon and Eriksen 2023) and schools (Schulze et al. 2020), as well as accentuating already present social vulnerabilities (Paveglio et al. 2015; Palaiologou et al. 2019). This theme is explored to a moderate extent in our research (8%). In events such as the 2018 Camp Fire in California, entire neighbourhoods were lost, including important community-based infrastructure such as schools and medical centres (Schulze et al. 2020; Hamideh et al. 2022). These spaces were relied upon more heavily by vulnerable groups, including elderly people and people with disabilities. Such spaces also often performed interconnected services outside their specified role, such as providing food and safe environments (Hamideh et al. 2022). The breakdown in community relationships through the destruction of key buildings offering social services can in some cases be difficult to reinstate, due to their interdependencies, and the potential relocation of people after a fire (Hamideh et al. 2022). Post-fire relocation has been documented to occur in different communities, and can accentuate feelings of loss, both for the individual and the community (Kulig et al. 2013; Schumann et al. 2020; Hamideh et al. 2022). Post fire relocation is often exacerbated by the exorbitant cost of re-building after wildfire events, which are particularly prohibitive if insurance is not in place or if a home is underinsured (Mockrin et al. 2015; Chase and Hansen 2021).

The evidence that wildfires can highlight inherent inequality within social systems, is exemplified by research undertaken in the USA, indicating communities of colour are often more vulnerable to wildfire impacts compared to other census tracts (Davies et al. 2018). Disadvantaged areas and Indigenous populations were also proportionally more affected than average within the 2020/21 Black-Summer fires in Australia (Nolan et al. 2021). These examples show that wildfires can highlight and perpetuate social suffering already present, disproportionately impacting the poorest and marginalised members of society. This disproportionate suffering from wildfires is also mirrored within other disasters, and has been widely discussed within the field of disaster sociology, indicating the extent to which suffering is unequal across society (Perry 2018). Whilst social suffering takes place within a group setting, the loss of such structures can have also significant cascading impacts on individuals, having further repercussions for mental and physical health of the affected populations.

### Mental suffering

Mental suffering can occur prior to an event starting (wildfire anxiety), particularly in areas with high fire risks and the appropriate climatic conditions, which are out of individuals’ control. During a fire, feelings of anxiety are often documented, for example when choosing whether to evacuate or to ‘stay and defend’ (Strahan and Watson 2019). Grief can occur as the fire spreads, particularly for those who can observe in real time the loss of something close to them; a home or a place of cultural significance for example. After a fire, mental suffering does not recede with the flames, and there is evidence to indicate that it can in fact get worse (Hrabok et al. 2020). Many studies have indicated that the trauma of a wildfire event can catalyse mental health disorders such as PTSD and depression (Silveira et al. 2021; Humphreys et al. 2022). The initial feeling of grief and worry can develop into long-term clinical diagnoses for people who have been impacted by a wildfire, which can become difficult to treat (Hrabok et al. 2020). First responders are not immune to this suffering: firefighters can also suffer significantly with the mental repercussions from wildfires and this will likely only get worse as fire regimes change around the world (Stanley et al. 2015; Singer 2021; Wolffe et al. 2022; Zhang et al. 2022). Mental suffering after a wildfire is evidently apparent in our review, but not to a large extent (3%). This highlights a need for more attention to the short- and long-term consequences of extreme wildfire events on mental suffering.

### Cultural suffering

When observing suffering that occurs within human populations, it is important to recognise that cultures can also suffer from the effects of wildfire. Whilst poorly covered in our literature review (0.8%), it is essential to acknowledge the consequences of wildfire within this sphere. In areas such as the USA, Canada and Australia, Indigenous communities often suffer culturally with the presence of wildfires, particularly when the events reach an extreme level. For example, the 2019/20 Black-Summer fires in Australia destroyed large areas of cultural significance to the local Aboriginal people. This caused suffering on a spiritual level (Williamson et al. 2020; Nolan et al. 2021; van Leeuwen and Miller-Sabbioni 2023). In California, the change in species composition after wildfire events can negatively affect elements of Indigenous life such as traditional food resources in forest-oak ecosystems (Voggesser et al. 2014). It is not just the presence of fire and changes in fire regimes that can cause suffering, but also the absence of fire. Fear of wildfires from outside settlers and the consequent ban of cultural burning in the USA caused suffering to Indigenous groups used to living with fire. This fire exclusion policy prevented some cultural actions, such as basket-weaving, from happening (Norgaard 2014; Adlam et al. 2021; Long et al. 2021). Wildfires also cause cultural suffering also outside Indigenous groups. Extreme wildfires in Portugal (2017) damaged cultural heritage assets, such as rock art and important parish houses, which were important for cultural gatherings (Figueiredo et al. 2021). Heritage areas that preserve native flora and fauna can also suffer considerably from wildfires, and these can struggle to recover with changes in fire frequency and severity (Laidlaw et al. 2022; Smith and Smith 2022). Despite the acknowledgement that cultural suffering can occur with wildfires, analyses of wildfire impacts often overlook such suffering and this requires further recognition.

### Resource suffering

Resource suffering considers wildfire impacts on resources predominantly utilised by human populations. Although not widely covered within the review (7%), essential infrastructures for daily life are often adversely affected by wildfire. Safe drinking water availability can be restricted due to the increased presence of hazardous particulates, both in the short and long term (Hohner et al. 2019; Robinne et al. 2021) as well as loss of power to grey infrastructure such as treatment plants (Jenkins et al. 2017). Power supplies can also suffer, as more areas approve shut offs in times of high risk (Rhodes et al. 2021; Zanocco et al. 2021; Sharafi et al. 2022). Transmission lines may also cause suffering through structural failure and consequent wildfire ignitions under dangerous weather conditions (Bliss et al. 2022). Supply chains including resources such as timber, or other vegetative outputs (e.g. wine grapes), can be adversely affected wildfire and wildfire smoke, having a cascading effect on other markets which rely on such products (Prestemon et al. 2006; Stephenson et al. 2013; Felipe et al. 2021; Summerson et al. 2021). Much of the suffering occurring from a resource perspective is documented by the economic impact, including for extreme fires (Meier et al. 2023), health-related costs (Limaye et al. 2019), suppression costs (Hope et al. 2016), and the reduction in services such as tourism (Kim and Jakus 2019). Carbon stocks and consequent CO_2_ emissions are likely to be negatively implicated by extreme wildfires. For example, burned areas are unable to sequester as much carbon, alongside further emissions of carbon within wildfire smoke (Campbell et al. 2007; Mackey et al. 2013; Ponomarev et al. 2021). Thus, the extensive resource suffering explored here can have implications within a wide range of contexts; affecting employment opportunities across supply chains, health of affected populations, and standards of living; indicating its interconnectedness with other elements of suffering.

### Positive impacts from wildfires

Exploring the six themes of suffering from wildfire events indicates fire’s potential to result in significant negative impacts. However, it is well acknowledged that not all wildfires are bad, and provide key processes in some socio-ecological systems, such as traditional rural livelihoods in mediterranean Europe (de Oliveira et al. 2023) and Indigenous communities, where cultural fire is integral to many food and medicine sources (Christianson et al. 2022). Even though positive impacts from wildfires were not explicitly sought after within our research set up, 10 of the papers reviewed acknowledged that whilst wildfires can cause suffering, fire is also often a positive component of ecosystems. Included within these papers were studies examining the positive impacts of fire on flora and fauna, including small mammals (Rollan and Real 2011), amphibeans (Lowe et al. 2013) and a variety of tree species (Battipaglia et al. 2016; Licht and Smith 2020). The use of fire was also indicated to have positive impacts on fuel loads (Starns et al. 2019) and forest carbon (Krofcheck et al. 2017). These positive outcomes from such fires evidence further that a lack of fire can also cause suffering, as explored within “Cultural suffering” Section.

## Interconnections: Suffering from a socio-ecological perspective

**Fig. 2:**
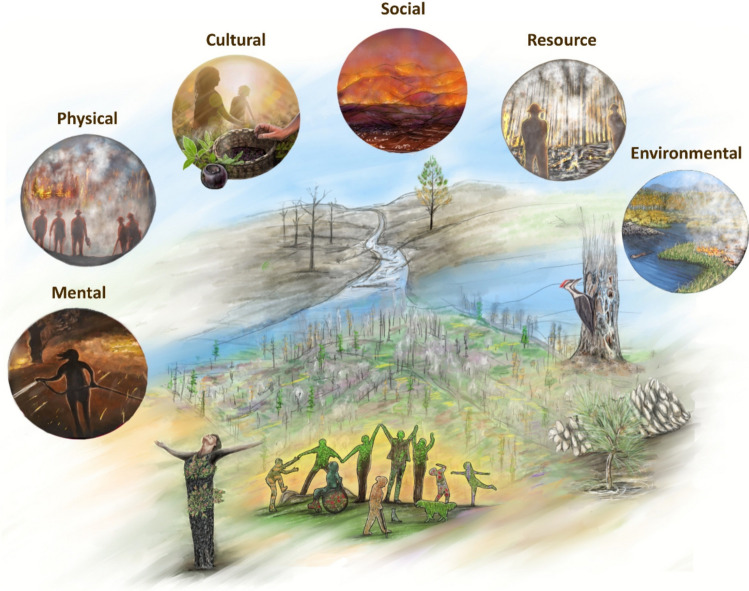
The six themes of wildfire suffering proposed, spanning across a broad range of contexts (artwork commissioned from Miriam Morell, Pyrosketchology)

Suffering from wildfires is evidently widespread on a multitude of spatial and temporal scales (Fig. 2). Whilst statistically, wildfires are not often associated with large-scale physical suffering such as high death tolls (Doerr and Santín 2016), these events can have far-reaching implications on individuals’ bodies, mental and emotional well-being, alongside the social systems, cultures and heritage which they develop and inhabit. The ecological environment can also suffer significantly both immediately after an event and in the years after (Lecina-Diaz et al. 2021; Burrell et al. 2022; Cowled et al. 2022). Alongside this, global systems such as supply chains, also experience suffering from wildfires (Ma et al. 2022), affecting accessible resources.

Particular interconnections exist between these themes of suffering. This invites a socio-ecological perspective that acknowledges the intertwined nature of anthropogenic and ecological systems (Folke et al. 2016). For example, environmental suffering affects social systems through knock on effects on resources, such as clean drinking water, and stable supply chains (Hohner et al. 2019; Felipe et al. 2021). Furthermore, the smoke released from wildfires (i.e. environmental suffering) likely has repercussions for anthropogenic well-being (Navarro 2020; Orr et al. 2020; Fernandes et al. 2022).

Post-wildfire, both environmental and anthropogenic survivors can often be said to recover. However, this often does not necessarily result in returning to the same state as before a fire. Mental health illnesses and physical injuries can linger months and years after an event, bringing difficulties in returning to ‘normal’ life for survivors (Brown et al. 2019; Grant and Runkle 2022). Ecosystem composition can be irreversibly altered due to fire regimes for which the inhabitants are unprepared (Vilà-Cabrera et al. 2012; Lecina-Diaz et al. 2021). Cultural artefacts destroyed in a fire will never return to what they once were (Nolan et al. 2021). These cascading impacts cross both social and ecological boundaries but seem inevitable in the aftermath of wildfire disasters recorded over the past decade. Here, it is pertinent to recognise that suffering is also expansive in other natural hazards that occur globally, such as floods (Jonkman and Vrijling 2008), landslides (Díaz et al. 2020) and drought (Gebremeskel et al. 2019). As with wildfires, these hazards can also be anthropogenically manipulated through, for example, changes in land use and anthropogenic migration. Thus, the framework and interconnections explored within this research has potential for examining such hazards, expanding the reach of these results beyond the topic of wildfire events.

Addressing suffering from such hazards starts by acknowledging it in all its diversity (Fig. 2). To counter the potential suffering explored in this research in regard to wildfires, pro-active investment in fire resilient landscapes and communities is required (Smith et al. 2016; Wunder et al. 2021; Newman Thacker et al. 2023), alongside adoption of adaptation actions that reduce the potential damages associated with a certain event (UNFCC 2022). Whilst it is impossible to remove all suffering associated with wildfires, both local and global strategies can prepare and react to wildfires. When exploring this framework alongside practioners and policymakers at organised wildfire-related events, we noticed that addressing the suffering that can result from continuing a business-as-usual approach can make the urgency of change tangible. As such, this suffering framework can be used as part of a visioning or futuring exercise, to guide developments of possible pathways to change (Kuiper et al. 2022; Uyttewaal et al. 2024). This framework could thus guide further projects surrounding adaptation to wildfires and integrated fire management, inviting a more equitable approach to these strategies. We illustrate this through two case studies, in which we identify prevalent suffering and highlight how adaptation strategies have impacted this suffering, or could have reduced further suffering.

### Las Maquinas Fire, Chile, 2017

The 2017 Las Maquinas complex in Chile offers an example the first ‘6th generation’ fire (Villagra & Paula 2021), spreading across more than 184.000 ha (Balocchi et al. 2020). A 6th generation fire behaves with such extreme intensity that firefighters cannot approach the fire and no suppression efforts are possible (Alcubierre et al. 2011; Villagra and Paula 2021). The fireline intensity within the Las Maquinas complex reached 113 000 kW, whilst the limit for indirect suppression is 10 000 kW, highlighting the intensity of the firestorms. Along with the Las Maquinas complex, the nation experienced a high frequency of concurrent extreme fires accumulating over half a million hectares, making it the worst fire season in recorded history.

From this megafire complex, the human death toll stood at eleven (Pliscoff et al. 2020). For such an extreme event, the physical suffering of civilians in terms of direct deaths and injuries was perhaps lower than expected. This can partially be attributed to the Chilean government’s investment into co-producing knowledge prior to the fire event after recognizing increased fire danger in the area. The National Forest Corporation (CONAF) and the California Fire Service (CALFIRE) signed a wide ranging collaboration agreement in 2016, with one element being that CONAF learned from CalFire’s techniques and strategies, but also including shared research between the countries (van Hensbergen and Cedergren 2021). The Chilean fire managers gained access to extensive and open access data, alongside highly trained personnel able to convert this data to valuable information to be used in decision-making.

CONAF’s ability to track the Las Maquinas fire complex meant that evacuations took place ahead of time (van Hensbergen and Cedergren 2021). Due to local community preparedness, evacuations were successful, and civilian death toll (physical suffering) remained low, with four deaths (van Hensbergen and Cedergren 2021). The Chilean Red Cross also made efforts to reduce mental suffering during and after the fire, offering psychological support to 400 families (IFRC 2018). Whilst this may have helped with the initial feelings of grief and loss, the support only lasted 1 month—possibly too short to alleviate mental suffering over longer time frames in the form of depression or PTSD (Brown et al. 2019). Seven firefighters also died during the Las Maquinas fires (physical suffering). Many of the firefighters tackling this event were volunteers, highlighting the risks associated with firefighting positions in the new era of megafires and that physical suffering is often borne directly by those on the frontlines. There is currently no data on the mental health status of the firefighters involved, nor the long-term effects of the fire on their physical health, despite evidence from other extreme fires that these effects can be substantial (Psarros et al. 2018; Navarro 2020). Whilst firefighting will always remain a hazardous occupation, the rise in such fires indicates vital steps must be taken to reduce these risks and alleviate the first responder suffering which has been recorded in the wake of other extreme fires (Singer 2021).

The intensity and size of the Las Maquinas fires can partly be attributed to the area’s domination by homogeneous pine plantations, backed by a centralised timber industry (van Hensbergen and Cedergren 2021). Whilst some prior discussions considered how to improve fire prevention and fire adapted landscapes in these plantations, they had not been implemented to any great extent (Bowman et al. 2019). The lack of integration of landscape planning and management techniques for wildfire led to little ecological resilience—the ecosystem had limited capacity to absorb such disturbances (Holling 1973) due to the forestry plantations homogenous nature and decrease in native tree species (Villagra and Paula 2021). Thus, the fire causes widespread environmental suffering. Significant amounts of livestock were killed and pine plantations devastated (van Hensbergen and Cedergren 2021). The fire easily leapt to structures, decimating entire towns such as Santa Olga (Bowman et al. 2019). For individuals, the loss of houses and communities caused significant mental, social and resource suffering, continuing to this day as the towns are rebuild (Villagra and Paula 2021). The contribution of this homogeneous and poorly managed landscape in powering this intense complex of fires is apparent (Bowman et al. 2019; Pliscoff et al. 2020). Had the landscape been more carefully adapted to wildfire disturbances, particularly the at-risk areas where communities intersected with forest plantations, both environmental and anthropogenic suffering may have significantly decreased.

Finally, the Las Maquinas complex exposed the fragility of the supply chain and economy. This resulted in widespread resource suffering. The area is a ‘commodity region’, focussing on the creation and exportation of a few specialised goods (timber and wine grapes) (Bustos-Gallardo and Prieto 2019). This reliance made the economic system vulnerable to wildfire risk. Despite this reliance there had been little policy or management put in place to make these commodities more resilient to wildfire (van Hensbergen and Cedergren 2021; Villagra and Paula 2021), thus many timber plantations and vineyards were eradicated or damaged beyond repair. This resource suffering also affecting global supply chains. On an individual scale, many plantation labourers experienced lower and more uncertain incomes due to these impacts, contributing to mental suffering long after the flames had receded (van Hensbergen and Cedergren 2021). Actions such as producing policies to support diversity in economic systems, alongside the aforementioned landscape planning, probably could have increased the resilience of these resources, and thus decreased the suffering of those reliant on them.

### Fort McMurray Fire, Canada, 2016

The 589 552 ha Fort McMurray fire was a severe event occurring in Alberta, Canada. It destroyed 2579 structures in Fort McMurray town (McGee 2019). No direct deaths were caused by the fire but two individuals perished in a vehicle accident (Mamuji and Rozdilsky 2019). With the large scale of the fire and the extensive urban area at risk, this lack of immediate physical suffering is particularly noteworthy and can be mostly attributed to the major evacuation effort, which took place as the fire raged. 88,000 people were evacuated from Fort McMurray (Adu et al. 2022). On a group scale and statistically, with the population remaining out of the way of direct harm, this evacuation is portrayed as “very successful” (Mamuji and Rozdilsky 2019). However, on the scale of an individual, surveys among survivors suggests significant emotional trauma alongside mental and cultural suffering (McGee 2019). Some of the population felt unprepared to evacuate and highlighted a lack of official warning, instead basing their decision to evacuate on environmental cues such as the visual proximity of the fire (McGee 2019). In some cases, participants actually stated that their perceptions of the fire risk were incorrect: they trusted social cues (such as neighbours not evacuating) to continue with ‘business as usual’ until the situation became urgent (McGee 2019). Other residents found the event traumatic and chaotic due to quick changes in instructions from official bodies (Thériault et al. 2021). Whilst people did adhere to the evacuation notices, these notices probably did not come in sufficient time for the participants to avoid mental trauma and suffering (Mamuji and Rozdilsky 2019). Furthermore, Indigenous members of the Fort McMurray community experienced suffering associated with the evacuation, losing cultural items such as ceremonial possessions, as well as being unable to carry out cultural traditions such as hunting and foraging once the flames had receded (Montesanti et al. 2021).

The unpredictability of the fire contributed to the short evacuation window and associated suffering. The fire front moved in ways often unexpected to the fire crews, demanding “skills and experience levels that are not normally present among initial attack crews or first responders” (MNP 2017). This highlights the need for fire services in areas of high risk to prepare for more convective driven fires (Castellnou et al. 2019), whereby the fire is driven by interactions with the atmosphere (Glenn et al. 2022; Grimm et al. 2022; Kirschner et al. 2023). Extreme fire behaviours driven by convection can be deadly, in particular due to their unpredictability and the formation of a pyrocumulonimbus (pyroCb) (Duane et al. 2021; Castellnou et al. 2022). The Fort McMurray pyroCB cloud produced its own lightning storm, resulting in further ignitions and driving extreme fire spread (Struzik 2017; Kovacs et al. 2019). Under these circumstances, it has been recognised that the low death toll (physical suffering) is remarkable, when compared with other such convective fires such as the Portuguese Pedrógão Grande fires (Mamuji and Rozdilsky 2019; Pinto et al. 2022). However, the toll on the environment (environmental suffering) was high, with ecosystems such as peat bogs (Wilkinson et al. 2018), as well as water and air quality being negatively impacted (Landis et al. 2018; Emmerton et al. 2020). The danger of convective fire behaviour highlights the essential need to understand their underlying processes in greater depth. By co-producing such knowledge, such as operational and research teams working together, this greater understanding may help avoid future fatalities.

Notably, the Fort McMurray area had tried to prepare for wildfire events through community empowerment, another adaptation strategy. The area forms part of the FireSmart program, which includes vegetation management, activities focusing on signage, education and the development of a Wildfire Mitigation Strategy (Mamuji and Rozdilsky 2019). Whilst it cannot be empirically judged what impact these actions had on the Fort McMurray fire, after the event the FireSmart program initiated building 520 ha of fuel breaks around at-risk urban areas.

The efficiency of this measure was likely influenced by the presence of the mitigation strategy prior to the fire (Mamuji and Rozdilsky 2019), as residents were already familiar with the scheme and its aims. This action shows how landscape management can often work concurrently with community empowerment to adapt areas to wildfire risks, and potentially alleviate suffering associated with future wildfire events. The adaptation actions explored here also indicate that the people of Fort McMurray are aware that wildfires are an ongoing risk that cannot be eradicated, and are thus making steps toward living with fire.

## Conclusions

Fire is irrevocably intertwined with fundamental social and ecological processes, and has helped facilitate human evolution for thousands of years (Glikson 2013). Wildfires, alongside anthropogenically caused traditional, cultural and Indigenous fires, have shaped many of the landscapes seen around the world today (McKenzie et al. 2011). Yet, in recent decades, devastating and destructive wildfires are affecting communities and ecosystems around the world, causing widespread suffering to both anthropogenic and natural environments. In this research, we apply Holdren’s (2008) concept that people must ‘mitigate, adapt or suffer’, initially coined by environmental-change science, to wildfire events. We explored literature surrounding suffering, impacts and effects of wildfires, establishing six themes of suffering:

- *Environmental*—impact on ecological processes, including soils, water, atmosphere, flora and fauna.
- *Physical*—impact on the human body, including those who die from wildfires, along with injuries, illnesses from smoke inhalation.
- *Social*—the effects of a fire on social processes, such as destruction of homes, schools and hospitals, along with negative impacts on community processes.
- *Mental*—suffering from mental health illnesses either catalysed or made worse by a wildfire event.
- *Cultural*—damage to areas or objects of cultural significance, alongside suffering caused by restrictions in cultural fire use and a consequent absence of fire.
- *Resource*—suffering associated with damage to systems such as water and power, as well as the destruction of economic goods.

It is important to acknowledge that not all fire is bad—and the absence of fire can produce suffering to cultures and ecosystems which are fire-dependent (Brotons et al. 2013; Christianson et al. 2022). Yet the wide range of individual and community suffering (Fig. 2) that can be caused by bad fire requires proactive measures that address the root causes of the current landscape fire challenge (AGIF 2023), rather than the typical predominant focus on fighting the flames. In response, this research has provided an integrated understanding of suffering embodied in wildfires, which may help direct triggers for change if paired with meaningful political action. We suggest the use of this suffering framework within visioning and futuring exercises, to guide developments of possible pathways to change. Concurrently, the Canada and Chile case studies illustrated how this framework of suffering can additionally guide adaptation principles. Such principles could help to proactively mitigate impacts of wildfire disasters, addressing suffering at individual level (physical, mental) as well as the damage to systems that can accentuate individual suffering (social, resource, cultural). This research highlights that mental and cultural suffering are significantly underrepresented in current research, illustrating the need to strengthen the field of fire social sciences (McCaffrey 2015). Consequently, future work could concentrate on reducing this inequity when aiming for holistic adaptation strategies that capture the complex interactions between these themes of suffering.

### Open research

The data used for the systematic literature review within this research is available in Supplementary information 2 in the form of an Excel spreadsheet.

## Supplementary Information

Below is the link to the electronic supplementary material.

Supplementary file1 (PDF 305 kb)

Supplementary file2 (XLS 1277 kb)
