# Supplementary material for: In this current wildfire crisis, acknowledge widespread suffering
Source: Ambio. 2025 Jan 28;54(5):759–73. doi: 10.1007/s13280-024-02105-5 (PMC11965086; doi:10.1007/s13280-024-02105-5)
Supplement: Supplementary file 1 — Supplementary file1 (PDF 305 kb) [file 13280_2024_2105_MOESM1_ESM.pdf]

Ambio

Supplementary Information

This supplementary information has not been peer reviewed

Title: In this current wildfire crisis, acknowledge widespread suffering

ROSES Protocol (from Haddaway et al. (2017))

1.0. Title
In this current wildfire crisis, acknowledge widespread suffering

2.0. Type of Review
Systematic Review Amendment
The initial review for this paper did not use an established protocol (eg ROSES/PRISMA) when first submitted to Ambio in April 2024. On recommendation of the editor reviewing the paper and of the two reviewees, all who stated extra information about the review process was needed, this protocol was created and the results of the review were slightly amended to stay in line with this systematic processing.

3.0. Authors' contacts
Fiona Newman Thacker, fiona.newman-thacker@wur.nl, Wageningen University
Kathleen Uyttewaal, kathleen.uytewaal@wur.nl, Wageningen University
Tomás Quinones, tquinones@technosylva.com , Technosylva
Rik Leemans, rik.leemans@wur.nl, Wageningen University
Bethany Hannah, bhannah@climateandwildfire.org , Climate & Wildfire Institute
Cathelijne R. Stoof, cathelijne.stoof@wur.nl, Wageningen University

4.0. Abstract
With climate change causing more extreme weather events globally, climate scientists have argued that societies have three options: mitigation, adaptation or suffering. In recent years, devastating wildfires have caused significant suffering, yet the extent of this suffering has not been defined. To encapsulate this suffering we determined impacts and effects of extreme wildfires through two systematic literature reviews. Six common themes of wildfire suffering emerged: environmental, social, physical, mental, cultural and resource suffering. These themes varied in scale: from local to regional; from individuals to communities; and from ecosystems to landscapes. We then applied these themes in the Las Maquinas (Chile) and Fort McMurray (Canada) wildfires. This highlighted several adaptation strategies that can reduce suffering, however our exploration indicates these strategies must address social and ecological factors. This analysis concludes that suffering from wildfires is diverse and widespread, and that significant engagement with adaptation strategies is needed if this is going to decrease.

5.0. Background

See main manuscript for the background rationale for this study.

Two systematic reviews were conducted, following a similar protocol, outlined in this document.

Review 1 focused on classifying suffering from wildfire events into themes using a narrative synthesis using literature specifically exploring suffering directly

Review 2 focused on exploring the themes created in Review 1 through a broader lens, classifying wildfire impacts and effects within the themes created in Review 1.

From here on, these reviews will be referred to as Review 1 and 2.

6.0. Stakeholder Engagement
The stakeholders involved in this review were researchers. The researchers formulated the research question within a workshop setting, as part of a training event within the EU-funded PhD programme PyroLife (insert weblink or cite the Living w Fire paper here). The workshop explored the concept introduced by Holdren (2001) that society will either mitigate, adapt or suffer in accordance with global climatic changes. For this review, research questions were designed aimed at applying and exploring this statement in association with wildfire events. A methodology was then chosen in the form of a

literature review after discussions between the researchers. The funding body of the research (EU) had no input into the content or methodology of the paper.

7.0. Objective of the Review

RQ1: What types of suffering are associated with wildfire events?
RQ2. How can the impacts of extreme wildfire events be studied more holistically through the use of suffering as an exploratory framework?

8.0. Deviations from Protocol

There are no deviations from this protocol.

9.0. Search Strategy

Database names: Web of Science, SCOPUS

Dates of Searching: May and June 2023

Institutional subscriptions to both databases provided by Wageningen University & Research.

No other evidence was utilised. No grey literature was utilised.

9.1. Search String

To investigate RQ1, Review One was conducted on Web of Science and SCOPUS using the search string: *wildfire suffering* between the dates 1990-2022.

To investigate RQ2, Review Two was conducted on Web of Science using two search strings: *extreme wildfire impacts* *extreme wildfire effects* between the dates 2013-2023.

9.2. Languages used

English

9.3. Estimating Comprehensiveness of the Search

The results of the search strings used for Review 1 and Review 2 were cross-checked with 13 articles which were identified at the initial workshop (described in section 6.0.) within which the idea for this article was created. In this exploratory phase of this research, searches were completed using Google Scholar to explore the base concept of suffering from wildfires, trying to establish what and who could suffer from such events. 8 out of these 13 articles were present within the review, shown in Table S1.

Title	Authors	Journal	Year Published	Included in Review
Cultural impacts to tribes from climate change influences on forests	Voggesser *et al.*	Climate Change and Indigenous Peoples in the United States (Book)	2013	Yes
Wildfires in Chernobyl-contaminated forests and risks to the population and the environment: A new nuclear disaster about to happen?	Evangeliou *et al.*	Environment International	2014	Yes
Physicochemical characterization of smoke aerosol during large-scale wildfires: Extreme event of August 2010 in Moscow	Popovicheva *et al.*	Atmospheric Environment	2014	Yes
Critical review of health impacts of wildfire smoke exposure	Reid *et al.*	Environmental Health Perspectives	2016	No
Defining extreme wildfire events: Difficulties, challenges, and impacts	Tedim *et al.*	Fire	2018	Yes
Amenities or disamenities? Estimating the impacts of extreme heat and wildfire on domestic US migration	Winkler & Rouleau	Population and Environment	2018	Yes
Preparedness and Experiences of Evacuees from the 2016 Fort McMurray Horse River Wildfire	McGee	Fire	2019	No
Health Effects of Climate Change-induced Wildfires and Heatwaves	Rosiello & Szema	Cureus	2019	Yes
Analysis of forest fire fatalities in Southern Europe: Spain, Portugal, Greece and Sardinia (Italy)	Molina Terrén *et al.*	International Journal of Wildland Fire	2019	No
Working in Smoke: Wildfire Impacts on the Health of Firefighters and Outdoor Workers and Mitigation Strategies	Navarro	Clinics in Chest Medicine	2020	Yes
Building Resilient Communities: The Traumatic Effect of Wildfire on Mati	Vallianou *et al.*	International Journal of Psychological and Behavioral Sciences	2020	No
Wildfire impacts on education and healthcare: Paradise, California, after the Camp Fire	Hamideh *et al.*	Natural Hazards	2021	No
Smoke emissions from the extreme wildfire events in central Portugal in October 2017	Fernandes *et al.*	International Journal of Wildland Fire	2022	Yes

Table S1: Thirteen articles illustrating the comprehensiveness of the search terms used in this review.

10.0. Article Screening and Study Inclusion/Exclusion Criteria

10.1. Screening Strategy

The articles were screened using the title and abstract of each paper. The title of the paper was evaluated to begin the determination surrounding the theme of the paper and its relevance to the study using the exclusion criteria listed in Section 10.2. The abstract of the paper was then used to clarify the theme and assess the papers appropriateness for inclusion within the review. If the study was to be excluded, it was categorised using the Exclusion Criteria.

If needed, there was also the potential to read the entirety of a paper within a third stage as part of this screening process. However, this was not necessary, as for each paper the aim and outcome of the paper was successfully delineated using the title and abstract.

10.2. Inclusion/Exclusion Criteria

The review was very broad in its aims, and was not investigating a particular discipline (e.g. environmental or social sciences), a particular study design (e.g. interviews or wildfire modelling) or focussing on a particular geographical area.

We did use restrictions in dates: Review 1 (1990-2022) and Review 2 (2013-2023). There were also restrictions in language (English). We used a shorter time period in Review 2 than Review 1 to focus on contemporary extreme wildfires.

The focus of this study was on papers looking specifically at the **impacts** of wildfire events over a variety of spatial and temporal scales. We therefore used exclusion criteria to filter the results, removing studies focusing solely on:

- Prediction of wildfires (risk assessment, ignition probability, wildfire spread modelling)
- Drivers of wildfires with no study of impact
- Evaluation of management techniques on wildfire outcomes
- Irrelevant temporal or spatial scale (paleofires, atomic behaviour, laboratory experiments)
- Evaluation of methodologies/technologies with no link to wildfire impacts
- Policy evaluation
- Impacts of other natural hazards/environmental risks (mention of wildfire but no direct study of wildfire impacts)
- Retraction or Comment on previous paper
- No discernible link to wildfires

11.0.Critical Appraisal Strategy

A critical appraisal strategy was not used for this review. The main reason for the decision not to critically appraise each article was due to the objectives of the review not being based in the outcome of the articles studied, but rather their overall focus in terms of subject matter. Secondly, as this review only used academic databases to search for relevant literature, each article will have undergone peer review to be published within their respective journals, thus decreasing the likelihood of less authentic research contributing to the results of this review.

12.0. Data extraction

12.1. Data extraction strategy

Review 1: The relevant data was downloaded from each database (Web of Science and SCOPUS) in CSV format, which was then converted to .xlsx format. 'Remove duplicates' within Excel was used to align the downloads from both databases and ensure there were no double counts of articles.

Review 2: The data extraction was similar to Review 1, however only Web of Science was utilised. Only one search engine was used for Review 2, due to the large amount of duplicates removed in Review 1 when using Web of Science *and* Scopus. It was therefore decided to concentrate the reviews singularly on Web of Science. Remove duplicates within Excel was used to compare the outputs from this data extraction to the results of Review 1. This was completed to ensure that any duplicate results across the two reviews were not classified twice, which may potentially skew the resulting classifications.

Data synthesis and presentation

12.2. Type of synthesis

Narrative synthesis

12.3.Narrative synthesis strategy

A narrative synthesis was deemed appropriate for the two reviews conducted to produce this systematic review. The narrative synthesis worked to identify trends within the included articles to comprehensively explore the impacts of wildfires and the ways in which suffering is displayed after a wildfire event occurs. These trends were not based on the empirical findings of the research

(quantitative or qualitative) but rather on the topic of the findings and the aims of the research endeavour. Therefore, the only appropriate synthesis strategy was to analyse the words of the extracted articles (narrative synthesis).

The narrative synthesis strategy was designed so that each article would be given an overall theme that matched the main suffering explored within the article in regard to wildfires. In Review 1, the narrative synthesis was designed to produce the initial themes, and in Review 2 the narrative synthesis aimed to categorise the outputs according to these predetermined themes.

14.0. Assessment of risk of publication bias

Results were only searched for in English, and so the utilised articles are likely to be exposed to an anglophone bias. However, English is the standard language of the international peer-reviewed literature. This review concentrated solely on outputs from databases searching registered peer-reviewed journals, and did not engage with grey literature. Therefore, we discern that the impact of only searching for literature in English is unlikely to have produced a significant bias within the results. Nonetheless, the decision to only engage with scientific literature is likely to produce a bias in its own right, as it is well known that Indigenous, local and traditional ecological knowledge and experiences are not well reflected within science. Therefore, this review is unlikely to reflect this knowledge to an accurate extent.

15.0 Demonstrating procedural independence

Because of the very high number of academic papers consulted (1468 in total), it was not feasible to contact individual authors to verify our classification of their work into the six themes of suffering.

16.0. Results

16.1. Number of search results

Review 1: SCOPUS: 49, Web of Science: 221, Total: 270

Review 2: 1198 (Web of Science only)

Total: 1468

16.2. Number of search results after duplicate removal

For Review 1, duplicates removed when compared against each database retrieval.

Articles remaining after duplicate removal from Review 1: 245

For Review 2, only Web of Science was utilised and so duplicates were removed if duplicated with articles from Review 1 only.

Articles remaining after duplicate removal from Review 2: 899

16.3. Full text screening exclusions

Full text screening was not part of this systematic literature review.

16.4. Title and abstract screening results

Results remaining after title and abstract had been examined using exclusion criteria.

Review 1: 108

Review 2: 425

The breakdown per exclusion category can be seen in Table S2.

Reason for Exclusion	Code	No. of Articles Excluded	
		Review 1	Review 2
Prediction of wildfires (risk assessment, ignition probability, wildfire spread modelling)	P	15	45
Drivers of wildfires with no study of impact	D	6	137
Evaluation of management techniques on wildfire outcomes	EM	9	41
Irrelevant temporal or spatial scale (paleofires, atomic behaviour, laboratory experiments)	S	4	19
Evaluation of methodologies/technologies with no link to wildfire impacts	MT	43	78
Policy evaluation	PE	1	9
Impacts of other natural hazards/environmental risks (mention of wildfire but no direct study of wildfire impacts)	NH	51	140
Retraction or Comment on previous paper	RC	2	0
No discernible link to wildfires	NW	7	5
**SUM**		**137**	**474**

Table S2: Displaying the exclusion criteria utilised for this review, alongside the associated code used to distinguish between exclusion criteria within the raw data files, and the number of files excluded for each criterion for each review.

16.5. Unobtainable articles

As full text screening was not an element of this review, no articles were unobtainable.

16.6. Narrative synthesis

The narrative synthesis of Review 1 resulted in five themes of suffering associated with the impacts of wildfire events. A sixth theme, positive impacts, was also noted. The breakdown of Review 1 per theme can be seen in Table S3.

Review 1 Included Articles		
Theme	*N*	*Percentage*
Environmental	71	67.62
Physical	10	9.52
Mental	3	2.86
Social	8	7.62
Resource	8	7.62
Positive	8	7.62

Table S3: the number of articles per theme as extracted in Review 1, alongside the percentage each theme is represented when compared against the sum of all articles in Review 1.

Using these themes, the results from Review 2 were themed accordingly, however a further theme had to be added (cultural suffering) to fully encapsulate the results of Review 2. Within Review 2 there were also examples of research articles which concentrated on the impacts of extreme weather events due to climate change, including wildfires. If this research was explicitly linked to extreme wildfire events and their impacts, it was included within the study. However, to distinguish them, these were counted separately to the articles focussing purely on wildfires. The breakdown of Review 2 can be seen in Table S4. For the theme 'positive', the two articles available referred specifically to extreme wildfires, with no articles exploring this theme through the lens of extreme weather events and climate change.

Review 2 Included Articles				
Theme	*Type*	*N*	*Percentage*	*Sum*
Environmental	Extreme Wildfire	247	58.12	*n*=256
	Climate Change	9	2.12	
Mental	Extreme Wildfire	9	2.12	*n*=11
	Climate Change	2	0.47	
Social	Extreme Wildfire	21	4.94	*n*=33
	Climate Change	12	2.82	
Physical	Extreme Wildfire	76	17.88	*n*=89
	Climate Change	12	2.82	
Resource	Extreme Wildfire	23	5.41	*n*=31
	Climate Change	8	1.88	
Cultural	Extreme Wildfire	4	0.94	*n*=4
	Climate Change	0	0	
Positive	Extreme Wildfires	2	0.47	*n*=2
	Climate change	0	0	

Table S4: showing the number of included articles within Review 2 and the percentage each theme is covered when compared against the sum of all articles.

16.7. Limitations of the Review

Searching for literature only in English gives an anglophone bias to the results and is likely to over represent Western countries. Furthermore, only the word ‘wildfire’ was used within the two reviews to target papers looking at such events, however other phrases, such as ‘wildland fire’ can sometimes be used to characterise these events, particularly in the USA. In Australia, the term ‘bushfire’ is often used in place of wildfire. As ‘bushfire’ was not included within the search terms of this review, this is a limitation of its scope. Furthermore, as mentioned in Section 14.0, the concentration of this review on scientific literature means it is likely that indigenous, local and traditional knowledge is underrepresented.

Only Web of Science was used for Review 2, therefore, research may have been missed if it was held singularly within other databases. However, the results from Review 1 showed a significant overlap between Web of Science and SCOPUS, and less relevant results from SCOPUS when using the exclusion criteria. This resulted in only 6 articles being included in Review 1 from SCOPUS. Therefore, it was asserted that the significant number of outputs from Web of Science for Review 2 was sufficient to explore the themes produced in Review 1.

Narrowing the search within Review 2 to concentrate on *extreme* wildfire events means that not every impact from all wildfires will have been captured. The justification for using extreme wildfire events is explained within the main methodology of the paper.

The timeframes explored within the two reviews were not exhaustive, limiting the results temporally. Extending the temporal reach of this review was outside the scope of this study, due to the significant amount of research outputs produced when combining both Review 1 and Review 2.

17.0.Conclusions

Fire is irrevocably intertwined with fundamental social and ecological processes, and has helped facilitate human evolution for thousands of years (Glikson 2013). Wildfires, alongside anthropogenically caused traditional, cultural and Indigenous fires, have shaped many of the landscapes seen around the world today (McKenzie et al. 2011). Yet, in recent decades, devastating and destructive

wildfires are affecting communities and ecosystems around the world, causing widespread suffering to both anthropogenic and natural environments. In this research, we apply Holdren's (2008) concept that people must 'mitigate, adapt or suffer', initially coined by environmental-change science, to wildfire events. We explored literature surrounding suffering, impacts and effects of wildfires, establishing six themes of suffering:

- **Environmental** – impact on ecological processes, including soils, water, atmosphere, flora and fauna.
- **Physical** – impact on the human body, including those who die from wildfires, along with injuries, illnesses from smoke inhalation.
- **Social** – the effects of a fire on social processes, such as destruction of homes, schools and hospitals, along with negative impacts on community processes.
- **Mental –** suffering from mental health illnesses either catalysed or made worse by a wildfire event.
- **Cultural –** damage to areas or objects of cultural significance, alongside suffering caused by restrictions in cultural fire use and a consequent absence of fire.
- **Resource** – suffering associated with damage to systems such as water and power, as well as the destruction of economic goods.

It is important to acknowledge that not all fire is bad – and the absence of fire can produce suffering to cultures and ecosystems which are fire-dependent (Brotons et al. 2013, Christianson et al. 2022). Yet the wide range of individual and community suffering (Fig. 2) that can be caused by bad fire requires proactive measures that address the root causes of the current landscape fire challenge (AGIF 2023), rather than the typical predominant focus on fighting the flames. In response, this research has provided an integrated understanding of suffering embodied in wildfires, which may help direct triggers for change if paired with meaningful political action. We suggest the use of this suffering framework within visioning and futuring exercises, to guide developments of possible pathways to change. Concurrently, the Canada and Chile case studies illustrated how this framework of suffering can additionally guide adaptation principles. Such principles could help to proactively mitigate impacts of wildfire disasters, addressing suffering at individual level (physical, mental) as well as the damage to systems that can accentuate individual suffering (social, resource, cultural). This research highlights that mental and cultural suffering are significantly underrepresented in current research, illustrating the need to strengthen the field of fire social sciences (McCaffrey 2015). Consequently, future work could concentrate on reducing this inequity when aiming for holistic adaptation strategies that capture the complex interactions between these themes of suffering.

18.0. Declarations

No competing interests to be declared.

Supplementary Material Reference List

AGIF. 2023. Landscape Fire Governance Framework Guiding Principles for Adjusting Strategies, Policies, and Management, to Global Change.

Brotons, L., N. Aquilué, M. de Cáceres, M. J. Fortin, and A. Fall. 2013. How Fire History, Fire Suppression Practices and Climate Change Affect Wildfire Regimes in Mediterranean Landscapes. PLoS ONE 8.

Christianson, A. C., C. R. Sutherland, F. Moola, N. Gonzalez Bautista, D. Young, and H. MacDonald. 2022. Centering Indigenous Voices: The Role of Fire in the Boreal Forest of North America. Current Forestry Reports 8:257–276.

Glikson, A. 2013. Fire and human evolution: The deep-time blueprints of the Anthropocene. Anthropocene 3:89–92.

Haddaway, N. ., B. Macura, P. Whaley, and A. Pullin. 2017. ROSES for Systematic Review Protocols Version 1.0.

McCaffrey, S. 2015. Community wildfire preparedness: A global state-of-the-knowledge summary of social science research. Current Forestry Reports 1:81–90.

McKenzie, D., C. Miller, and D. A. Falk. 2011. Toward a Theory of Landscape Fire. Pages 3–25 *in* D. McKenzie, C. Miller, and D. A. Falk, editors. The Landscape Ecology of Fire. Springer.
